# Fat embolism: a rare cause of pulmonary infarction

**DOI:** 10.36416/1806-3756/e20240113

**Published:** 2024-06-21

**Authors:** Cyro Antonio Fonseca, Gláucia Zanetti, Edson Marchiori

**Affiliations:** 1. Hospital Unimed Rio, Rio de Janeiro (RJ) Brasil.; 2. Universidade Federal do Rio de Janeiro, Rio de Janeiro (RJ) Brasil.

**Figure 1:**
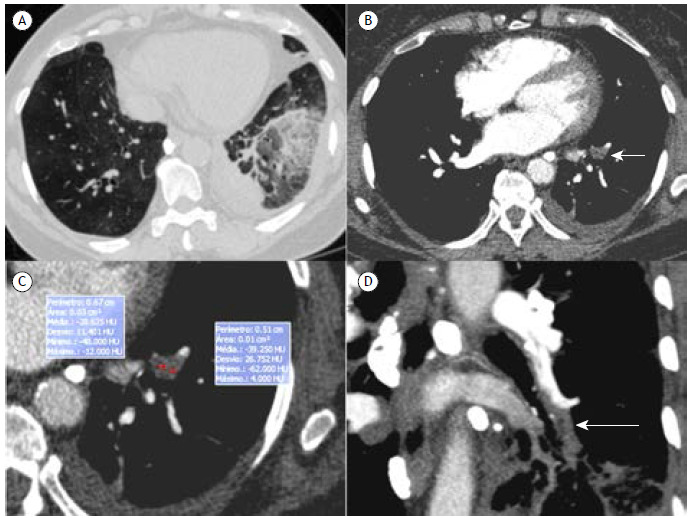
In A, unenhanced chest CT image (lung window) showing heterogeneous ground-glass opacities in the left lower lobe, with internal reticulation, compatible with pulmonary infarction. Note also the pleural reaction. In B, enhanced axial CT image showing a heterogeneous filling defect in the left lower-lobe artery (arrow). In C, magnified image of the emboli in B showing internal fatty density (-29 to -39 Hounsfield units). In D, coronal oblique reconstruction showing the heterogeneous emboli (arrow).

A 68-year-old woman was admitted to the emergency department with sudden dyspnea and chest pain of 3 days’ duration. She had had a domestic accident resulting in closed tibial and fibular fractures 5 days prior. Chest CT showed a filling defect in the left lower-lobe pulmonary artery with fatty attenuation, and a ground-glass opacity in this lobe, compatible with pulmonary infarction (Figure 1). The final diagnosis was pulmonary infarction due to macroscopic fat embolism.

Fat embolism syndrome, defined as the release of fat into the systemic or pulmonary circulation, is rare. It usually occurs after long bone fracture, orthopedic surgery, or cosmetic procedures.^1^ The majority of fat embolism cases are microscopic, presenting on CT as bilateral patchy or diffuse ground-glass opacities. Macroscopic fat embolism is a rare presentation of the disease in which macroscopic fat deposits are present in the pulmonary arteries. Its diagnosis is based on the demonstration of fat-attenuation filling defects in these arteries. Fat typically has negative attenuation values, enabling the distinction of fat embolism from pulmonary thromboembolism, characterized by positive attenuation values. This differentiation can have important implications for patient management.^1^ ^-^ ^3^
